# Shear stress and pathophysiological PI3K involvement in vascular malformations

**DOI:** 10.1172/JCI172843

**Published:** 2024-05-15

**Authors:** Salim Abdelilah-Seyfried, Roxana Ola

**Affiliations:** 1 Institute of Biochemistry and Biology, Potsdam University, Potsdam, Germany.; 2 Experimental Pharmacology Mannheim, European Center for Angioscience, Medical Faculty Mannheim, Heidelberg University, Mannheim, Germany.

## Abstract

Molecular characterization of vascular anomalies has revealed that affected endothelial cells (ECs) harbor gain-of-function (GOF) mutations in the gene encoding the catalytic α subunit of PI3Kα (*PIK3CA*). These *PIK3CA* mutations are known to cause solid cancers when occurring in other tissues. *PIK3CA*-related vascular anomalies, or “PIKopathies,” range from simple, i.e., restricted to a particular form of malformation, to complex, i.e., presenting with a range of hyperplasia phenotypes, including the *PIK3CA*-related overgrowth spectrum. Interestingly, development of PIKopathies is affected by fluid shear stress (FSS), a physiological stimulus caused by blood or lymph flow. These findings implicate PI3K in mediating physiological EC responses to FSS conditions characteristic of lymphatic and capillary vessel beds. Consistent with this hypothesis, increased PI3K signaling also contributes to cerebral cavernous malformations, a vascular disorder that affects low-perfused brain venous capillaries. Because the GOF activity of PI3K and its signaling partners are excellent drug targets, understanding *PIK3CA*’s role in the development of vascular anomalies may inform therapeutic strategies to normalize EC responses in the diseased state. This Review focuses on *PIK3CA*’s role in mediating EC responses to FSS and discusses current understanding of *PIK3CA* dysregulation in a range of vascular anomalies that particularly affect low-perfused regions of the vasculature. We also discuss recent surprising findings linking increased PI3K signaling to fast-flow arteriovenous malformations in hereditary hemorrhagic telangiectasias.

## Introduction

Vascular malformations are caused by genetic defects in endothelial cells (ECs) that line the interior of blood and lymphatic vessels. Most vascular anomalies arise during early vascular development and are characterized by abnormal life-long localized vessel growth. This can cause severe chronic pain, morphological deformations, bleeding, and even life-threatening conditions. Sporadically, vascular anomalies can also occur with a later onset.

For decades, vascular research had a strong focus on the identification of EC activation programs that drive developmental angiogenesis. Yet, more recent advances in vascular biology have demonstrated that, instead, fully differentiated and quiescent ECs are sensitive and responsive to physiological changes in their microenvironment and act as guardians of vascular homeostasis (1–3). These discoveries are highly relevant for understanding the etiology of vascular anomalies, some of which are recognized as resulting from disrupted developmental programs, while others result from a localized loss of EC quiescence of fully differentiated vessels. Loss of EC quiescence triggers a weakening of cellular junctions and promotes proliferation, ultimately leading to vascular dysfunction and disease progression. Therefore, identifying and understanding what regulates the balance between EC activation and quiescence is a high priority in the field of vascular biology.

The search for factors that trigger lesion formation led to the discovery that an overactivation of PI3K signaling plays a central role in the disease etiology of many types of vascular anomalies (summarized in Table 1). PI3K overactivation can be caused either through genetic gain-of-function (GOF) mutations in the gene encoding its catalytic α subunit PI3Kα/p110α (*PIK3CA*) or upon a physiological overstimulation by upstream regulators. The PI3K family of lipid enzymes consists of eight distinct catalytical isoforms grouped into three major classes based on their structure and substrate preferences. When stimulated, class I family members PI3Kα, PI3Kβ, PI3Kδ, and PI3Kγ phosphorylate phosphatidylinositol-4,5-bisphosphate (PIP2) to phosphatidylinositol-3,4,5-trisphosphate (PIP3). Genetic targeting of PI3K isoforms revealed that PI3Kα is essential for development of the blood and lymphatic vasculature (4, 5) and indispensable for the acquisition of a venous fate during vasculogenesis (6). The PI3Kβ isoform plays a role in ischemic heart disease by restricting VEGF-induced PI3Kα/AKT activation (7), and PI3Kδ is associated with inflammatory processes (8). Among class II family members, PI3K-C2α regulates vesicular trafficking, which has a crucial role in vascular development and homeostasis (9). The PI3K-C2α isoform is also involved in primary cilium function (10), suggesting that there is some involvement of this protein with EC responses to fluid shear stress (FSS). Recently, the class II PI3K-C2β isoform was shown to restrict activation of mTORC1-mediated EC size during angiogenic growth (11). Functions of PI3K-C2γ and of the only class III isoform, Vps34, in ECs have not yet been documented.

The production of PIP3 at the plasma membrane by class I PI3Ks results in an activation of AKT protein kinases (12, 13). While the three AKT isoforms display redundant roles during embryonic vascular development (14), the endothelial isoform AKT1 is essential for maintaining vascular integrity (15). AKT acts upstream of forkhead box O (FOXO) family transcription factors (16) that promote cell survival and metabolic reprogramming via cMYC (17) or mTOR (18, 19), which initiates protein synthesis. PI3K/AKT represents a major signaling hub that determines EC fate as well as EC growth, migration, actin cytoskeleton remodeling, metabolism, and vesicular trafficking. PI3Kα exerts functions also beyond AKT and regulates actomyosin contractility by fine-tuning the activity of the myosin light chain through activation of the small GTPase RhoA and the myosin light chain phosphatase (5, 20, 21).

This research points at PI3Kα as a central player in the development, homeostasis, and integrity of vessel beds, providing new angles for therapeutic interventions in a wide range of vascular anomalies associated with an overactivation of this kinase (22–27). The discovery that PI3Kα predominantly affects low-perfused vessel beds hints at its homeostatic role in ECs situated in regions of the blood and lymphatic vasculature that experience only low levels of FSS.

**Figure 1:**
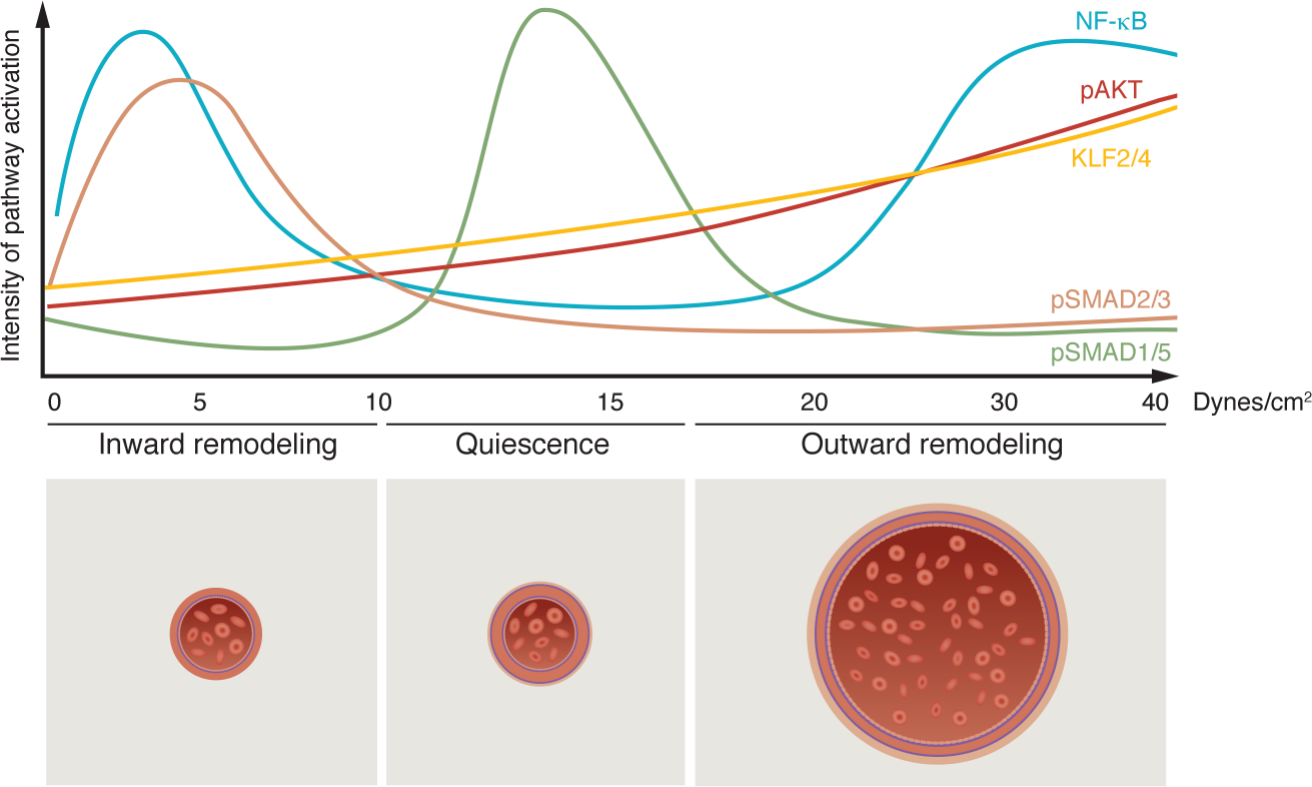
Laminar FSS regulates the balance of EC remodeling and quiescence. The magnitude of FSS (dynes/cm^2^) activates/suppresses divergent signaling pathways, promoting the remodeling versus quiescence of ECs. This involves varying intensities of TGF-β (pSMAD2/3), BMP (pSMAD1/5), NF-κB, KLF2/4, and pAKT pathway activation. This notion has been reinforced by the finding that defective sensing of FSS by ECs disrupts vascular homeostasis.

The role of mechanobiology in the development of vascular anomalies has raised much interest. FSS resulting from blood flow against the vessel wall exerts mechanical forces upon ECs. Mechanical transduction of FSS triggers biomechanical signaling and transcriptional changes, which maintain ECs in a quiescent state and ensure a healthy physiology of vessel beds (reviewed in refs. 28–33). Several biomechanical signaling pathways have been implicated in FSS sensing, including the PI3K/AKT/mTOR, RAS/MAPK, NOTCH, TGF-β/BMP, NF-κB, MEKK3/ERK5/KLF2/4, and HIPPO pathways (reviewed in refs. 34–40). The genetic and molecular identification of these divergent signaling pathways led to the realization that they control the maintenance of EC quiescence in different regions of the vasculature that are exposed to characteristic and distinct levels of FSS (Figure 1). This has also reinforced the concept that defective FSS sensing and responses in ECs disrupt the homeostasis of vessel beds, thus contributing to vascular anomalies.

This Review discusses the critical physiological role of PI3K in mediating EC responses to FSS in healthy vessels. We address recent advances in understanding how increased PI3K signaling, resulting from either genetic mutations or nongenetic overstimulation, affects the etiopathology of a continuum of molecularly related vascular anomalies that arise in regions of the vasculature that are characterized by slow fluid flow. These include venous malformations (VMs) and cerebral cavernous malformations (CCMs). We also highlight the surprising link between PI3K signaling and the development of arteriovenous malformations (AVMs), considered fast-flow pathologies of the vasculature, that occur in hereditary hemorrhagic telangiectasia (HHT) and other contexts. The role of PI3K in the development of lymphatic malformations has already been the focus of a number of excellent reviews and will not be discussed here.

## PI3K/AKT mediates EC responses upon FSS

**Figure 2:**
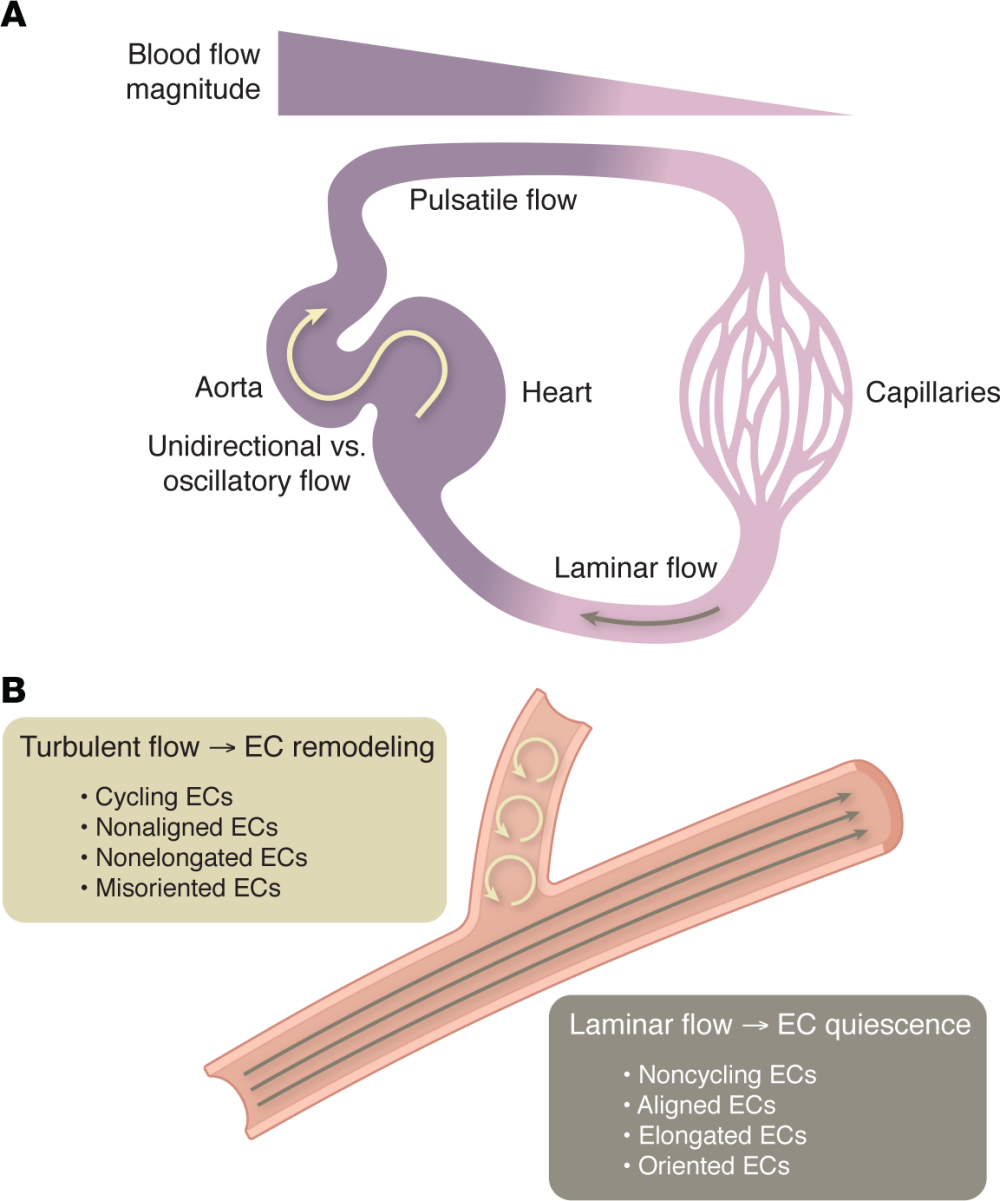
Different parameters of FSS and vascular morphology affect EC. ECs are characterized by an intrinsic capacity to rapidly adapt and respond to the mechanical stimulus exerted by FSS. (**A**) FSS varies with respect to the magnitude, which refers to its maximal force, and the flow pattern, which can either be pulsatile or laminar and which can diverge with respect to its direction (unidirectional or bidirectional/oscillatory). (**B**) Turbulent flow patterns can arise at branch points or due to shifts in the ratio between inertial and viscous forces.

ECs are characterized by an intrinsic capacity to rapidly adapt and respond to the mechanical stimulus exerted by FSS. This response can affect their proliferation, shape, movement, and cell fate. FSS varies with respect to its magnitude, which refers to its maximal force, and its flow pattern, which can be laminar, pulsatile, or turbulent and which can diverge with respect to its direction (unidirectional or bidirectional/oscillatory) (Figure 2). Fluid flow patterns can change from laminar to turbulent based on shifts in the ratio between inertial and viscous forces. In fluid mechanics, this ratio is expressed by the Reynolds number. Large blood vessels are characterized by a high Reynolds number and are more prone to exhibit turbulent flow. Laminar flow patterns prevail at low Reynolds numbers that are characteristic of small capillaries. Importantly, the Reynolds number can also rise in small capillaries when fluid flow drops to a low magnitude. This predicts that a turbulent flow pattern can also occur in small vessels.

Consequently, in different regions of the vasculature that are exposed to distinct FSS patterns, the responses of ECs differ and can be homeostatic, adaptive, or maladaptive. A laminar and unidirectional FSS pattern will induce EC responses that favor quiescence and vascular stability, whereas a disturbed flow pattern, arising in regions of the vasculature with branching points or curvatures, may trigger EC proliferation and a gene expression profile characteristic of inflammation and oxidative stress (41–43). A laminar flow pattern of a high magnitude will promote an elongation and alignment of ECs parallel to the direction of flow; it will also trigger their orientation and migration against flow (collectively termed axial polarity-mediated reverse migration). By contrast, in capillaries exposed to only a low magnitude of flow, ECs fail to align or to initiate polarity-induced migration (44–48). This response prevents EC regression in slowly perfused vessel beds. The response of ECs to FSS also affects vessel diameter (49–52). This capacity of ECs to respond to different levels of fluid flow has been attributed to the existence of a “FSS set point.” The FSS set point is a concept positing that ECs “memorize” an optimized FSS and that they will respond by inward or outward remodeling of the vessel diameter if transient alterations of FSS levels occur (53, 54) (reviewed in refs. 33, 55, 56). Therefore, small changes in physiological flow patterns drive adaptive and transient EC remodeling of the vascular wall until the original FSS set point is restored. It is also conceivable that a defective signal transduction triggered by FSS will result in maladapted and chronic vessel remodeling, thus contributing to vascular instability and disease.

**Figure 3:**
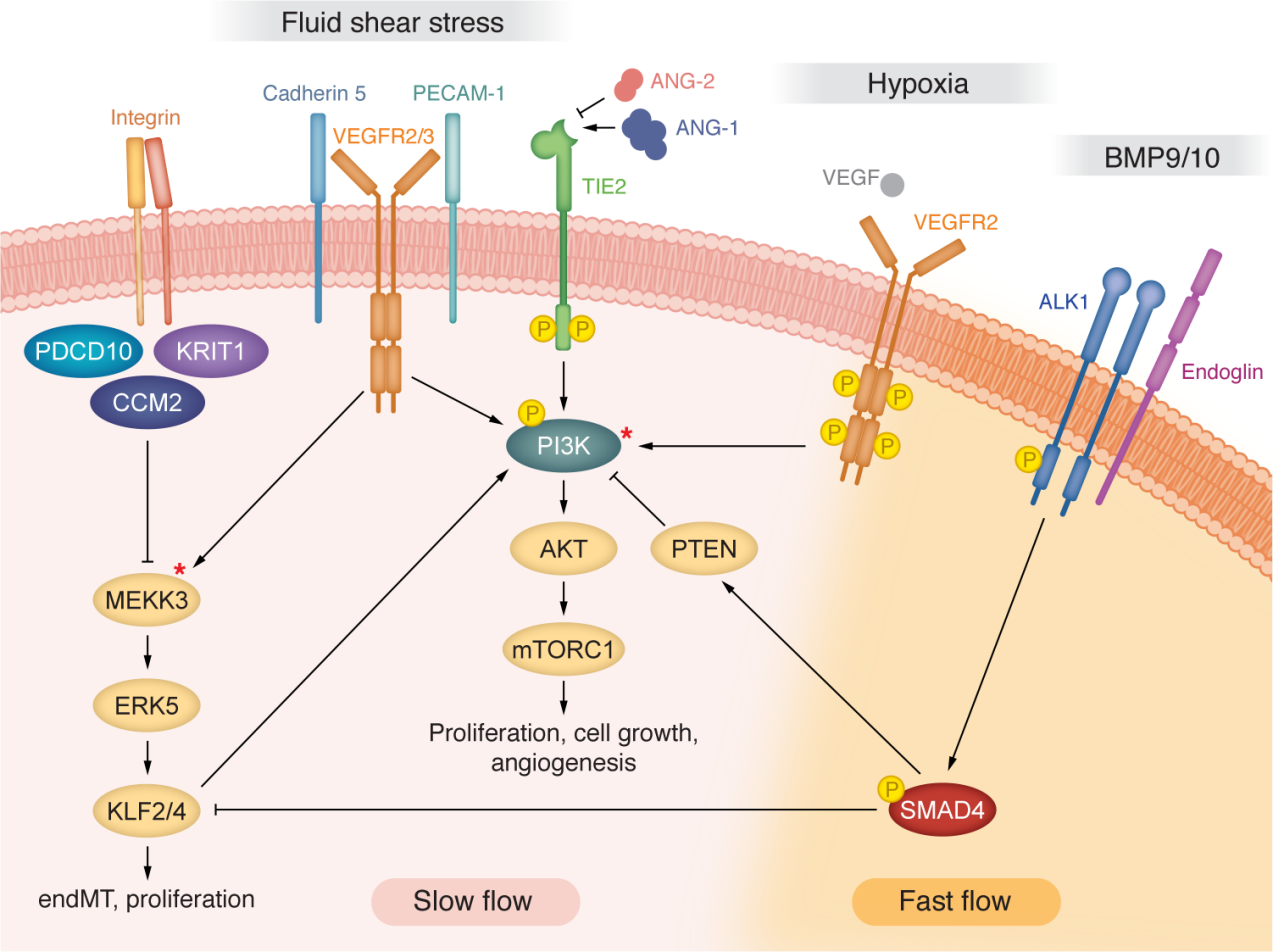
Interconnected signaling pathways in vascular anomalies involving PI3K signaling. Fluid shear stress (FSS) activates mechanosensitive signaling, involving, among others, a FSS set point machinery comprising VE-cadherin (cadherin 5), VEGFR2/3, and PECAM-1, which activates downstream PI3K signaling. FSS also triggers an activation of the MEKK3 pathway. In CCM, an overactivation of KLF2/4 in small venous capillaries triggers a pathological endothelial-mesenchymal transition (endMT) and overproliferation. Asterisks indicate proteins for which oncogenic GOF mutations have been identified in slow-flow vascular anomalies. Increased PI3K signaling characterizes slow-flow venous and cavernous malformations but also HHT-related fast-flow AVMs caused by LOF mutations in genes encoding ALK1, endoglin, BMP9, or SMAD4.

Functional experiments in mice, zebrafish, and human ECs led to the identification of various molecular components that mediate FSS responses. Several of these studies point to a central role of PI3K signaling directly downstream of a FSS set point machinery, which assembles around EC adherens junctions and comprises PECAM-1, vascular endothelial–cadherin (VE-cadherin), and the VEGF receptors VEGFR2 and VEGFR3 (57–60) (Figure 3). Recent evidence suggests that PECAM-1 acts as a relay in the FSS cascade downstream of other mechanosensors, including GPCRs and mechanically gated ion channels, among others (61–63).

Another FSS mediator complex upstream of PECAM-1 involves PlexinD1, which becomes engaged in a receptor complex with Neuropilin 1 and VEGFR2; this triggers a ligand-independent activation of VEGFRs and initiates a shear stress signaling cascade involving PI3K and its central downstream mediator AKT. The activation of AKT is dependent upon the magnitude of FSS (64). BMP9 circulating in blood acts upstream of FSS to limit excessive PI3K/AKT activation (65). Yet, the precise mechanism, i.e., how activation of AKT is balanced, remains to be elucidated. In static cells, BMP9 inhibits activation of PI3K/AKT through a negative feedback loop that involves transcriptional repression of casein kinase 2–mediated (CK2-mediated) dephosphorylation of the phosphatase and tensin homolog (PTEN) (65). PTEN is a phosphatase that negatively regulates PI3K activity. Dephosphorylated PTEN binds the cell membrane and reduces the production of PIP3, thus antagonizing PI3K/AKT signaling. BMP9 also restricts the VEGFA-induced activation of PI3K/AKT, which involves PTEN as a repressive factor (66). Interestingly, laminar FSS represses PTEN levels in a time- and magnitude-dependent manner, while AKT activation follows the exact opposite trend (67). Thus, PTEN downstream of BMP9 and FSS or angiogenic stimuli restricts PI3K activity.

The activation of PI3K/AKT signaling by physiological FSS regulates EC alignment (68), and this is also important for the activation of NOS3-mediated vasodilation (69) and for cell survival (70). Thus, the alignment of ECs in response to laminar FSS is mediated by an activation of PI3K and is part of an antiinflammatory and vasodilatory adaptation program that is characteristic of healthy vessels. SMAD4 mediates many EC quiescence events, as mentioned above, and this requires a precise threshold of PI3K/AKT activity (64). Therefore, PI3K emerges as a signaling hub in several pathways that are activated in response to FSS, and accurate levels of PI3K activation ensure a correct balance between EC activation and quiescence (Figure 3).

PI3K’s key positioning in the FSS response is also linked to its paramount roles in signaling downstream of several growth or angiogenesis receptors. These include the VEGFR2 and VEGFR3 receptors that are activated during vascular growth and remodeling by FSS, hypoxic physiological conditions, or proangiogenic factors, among others (reviewed in ref. 71). PI3K also mediates TIE2 (encoded by *TEK*) receptor signaling upon binding of angiopoietin-1 (ANG1) (72). Signaling by ANG1/TIE2 maintains vascular quiescence in the murine adult vasculature (73–75). In part, this effect is mediated by the transcriptional regulator Krüppel-like factor 2 (KLF2), which is among the downstream targets activated by ANG1/TIE2 signaling in ECs (76), and this activation requires PI3K (72). KLF2 and KLF4 are transcriptional regulators that are activated by FSS (77–84) and trigger gene expression that promotes vascular quiescence (43, 78, 83, 85–87). *TEK* itself is upregulated upon laminar FSS in a KLF4-dependent manner, and TIE2 is required together with PECAM-1 for FSS-induced AKT activation (64, 88). Altogether, these studies suggest that the development and the maintenance of a mature vascular network is guided by both mechanical stimuli triggered by FSS and/or physiological conditions such as the availability of oxygen and nutrients. These factors will trigger biomechanical and biochemical signaling within ECs that both impinge upon PI3K/AKT activation. Therefore, PI3K is a key factor in the development and maintenance of vascular homeostasis in the blood and lymphatic endothelium.

## Pathological PI3K signaling in vascular anomalies

In recent years, genetic studies of vascular anomalies have led to the identification of mutations that impinge upon aberrant activation of the PI3K pathway (GOF mutations in *TEK*, *PIK3CA*, or *AKT* and loss-of-function [LOF] mutations in *PTEN*) (reviewed in ref. 89). These findings underscore the crucial role of this pathway in vascular integrity and homeostasis. A recent study identified somatic LOF mutations in *PIK3R1* and GOF mutations in *PIK3CA* that were associated with a novel vascular malformation termed capillary malformation with dilated veins (CMDV) (90).

Activating mutations in *PIK3CA* and *AKT* are predominantly somatic in nature, as germline mutations in these genes are incompatible with survival. Similar to *PIK3CA*, somatic mutations in *AKT* may also manifest as isolated lesions or within a complex syndromic phenotype, the Proteus syndrome (91). In contrast, *TEK* mutations can occur either somatically or in the germline. A large spectrum of LOF *PTEN* mutations is frequently inherited via the germline and leads to PTEN hamartoma tumor syndrome (PHTS) (92, 93).

Increasing evidence suggests that hemodynamic forces due to FSS strongly contribute to the occurrences of many types of vascular anomalies. Both the PI3K/AKT/mTOR and the RAS/MAPK pathways, together with the NO signaling pathway, become excessively activated in different types of vascular malformations (reviewed in refs. 22, 37). The same exact pathways are also stimulated by physiological laminar FSS, which transiently activates signaling via MAPK (reviewed in ref. 94), Rho family GTPase (reviewed in ref. 95), and NO signaling pathways (NOS3 and NOS), with a release of NO, an important antiinflammatory and vasodilatory signal associated with healthy vessels (96, 97). FSS can also trigger more permanent effects via ERK5, driving KLF2/4 transcription factor activation that causes an antiinflammatory phenotype (35, 80). Strikingly, disturbed flow activates the same pathways, yet also activates ROS and NF-κB proinflammatory modulators (98–100). These pathways link biomechanical signaling in response to FSS with key processes of EC biology such as cell fate determination, cell orientation and migration, and cell proliferation.

The elongation and alignment of ECs is mediated by cytoskeletal remodeling driven by the Rho GTPase family proteins RHO, RAC, and CDC42, whose activities are determined by FSS (101–104). Based on their occurrences in distinctly perfused vessel beds, a large range of vascular malformations can be categorized as either slow-flow “PIKopathies” that involve the PI3K/AKT/mTOR pathway or fast-flow “RASopathies,” involving a stimulation of the RAS/MAPK pathway (reviewed in ref. 22). This suggests critical roles of PI3K and RAS as key mediators for controlling FSS-dependent vascular biology. In alignment with such divergent physiological roles, both factors are also important fate determinants for venous versus arterial cell identities, respectively (6, 105–108). The maintenance of arterial identity also depends on FSS-mediated cell cycle arrest, regulated by the NOTCH and canonical BMP9/10 signaling pathways (64, 109). Interestingly, the class I isoform PI3Kβ binds to RAC1 and CDC42 via its RAS-binding domain, indicating that these two signaling hubs are interconnected (110).

The (patho-)physiological role of PI3K within slow-flow regions of the vasculature has been particularly well-characterized. The molecular characterization of an entire spectrum of slow-flow vascular malformations revealed the presence of well-known oncogenic driver mutations in *PIK3CA* that cause cancer in other tissues (111) and drive venous and lymphatic malformations in the endothelium (112–118). Therefore, vascular lesions in slowly perfused vessels beds have been linked to excessive PI3K signaling caused by either genetic GOF mutations or upon a physiological overstimulation of this pathway that converts into pathological signaling. Increased PI3K/AKT activation causes venous, lymphatic, and/or capillary ECs to lose quiescence and undergo persistent remodeling. These findings suggest that PI3K acts as a rheostat in controlling EC responses to FSS and that this pathway is particularly sensitive to slow FSS conditions.

## Genetic third hits involving *PIK3CA* in CCMs

Much of our understanding about the role of biomechanical responses in vascular malformations has come from work on CCMs. CCMs arise after a biallelic loss of one of three *CCM* genes encoding CCM1 (also known as KRIT1) (119), CCM2 (120), or PDCD10 (referred to herein as CCM3) (121). These mutations can cause the formation of vascular malformations that arise mainly within slowly perfused venous capillaries of the brain vasculature and result in bleeding with unpredictable consequences for patients. Molecular and genetic studies in different CCM animal models and human ECs revealed that a direct physical interaction of CCM2 with MEKK3 kinase (encoded by *MAP3K3*) prevents an activating phosphorylation (122, 123). MEKK3 stimulates signaling via ERK5 and the downstream transcriptional regulators KLF2 and KLF4 (123–127). The stimulation of high expression levels of KLF2/4 within brain capillary ECs contributes to CCM lesion formation (124, 127, 128). Suppression of the MEKK3/ERK5/KLF2/4 pathway prevented the formation of disease phenotypes in CCM models (124, 125, 127, 129). This research established the CCM proteins as gatekeepers of appropriate biomechanical signaling responses within slowly perfused vessel beds and established their role in suppressing an overactivation of pathological KLF2/4 signaling under conditions of low FSS (Figure 3).

Yet, the discoveries of the disease-associated *CCM* genes soon came with the realization that a monogenic trait was not sufficient to explain the natural disease progression in humans and that other “third hits” may be involved (reviewed in ref. 130). Such third hits involve truly genetic changes or may be nongenetic in nature. Sequencing studies of human CCM lesions revealed that, indeed, genetic third hits occur in CCM lesions. Many human CCM tissue samples harbored the same oncogenic *PIK3CA* GOF mutations that also occur in cancer and in other vascular anomalies (24, 131). This finding suggested a surprising genetic link between the CCM/MEKK3/KLF2/4 and the PI3K/AKT/mTOR pathways in CCM lesion formation. In further support of such a model, genetic studies in patients with spontaneous forms of CCM revealed that an overstimulation of the MEKK3/KLF2/4 pathway due to the occurrence of oncogenic *MAP3K3* GOF mutations correlates with the formation of spontaneous CCM lesions (132–134). In sporadic nonfamilial forms of CCM, *CCM* LOF or *MAP3K3* GOF mutations were frequently found together with *PIK3CA* GOF mutations (134). Particularly aggressive forms of CCMs arise when both oncogenic *Map3k3* and *Pik3ca* GOF mutations co-occur within the affected murine endothelium (135). In CCM disease animal models and human CCM protein-deficient ECs, oncogenic *Pik3ca^H1047R^* mutations synergized with loss of CCM proteins and strongly aggravated lesion formation (24). This triggered a strong mTORC1 and S6Kinase activation, and treatment with the FDA-approved drug rapamycin (sirolimus), an mTORC1 inhibitor commonly used for the treatment of venous and lymphatic malformations caused by *Pik3ca* GOF mutations, improved lesion outcomes. The pharmacological suppression of mTORC1 by rapamycin also suppressed lesion formation in a murine model that combined inducible *Ccm* LOF and *Pik3ca* GOF mutations (136). These discoveries suggested that a cancer-like three-hit mechanism is involved in CCM lesion formation (24). According to that model, *CCM* genes act as vascular “suppressor genes” and homozygous loss of these genes leads to increased vessel growth. Excessive vessel overgrowth is then fueled when affected ECs acquire an additional third oncogenic driver mutation.

## Aberrant PI3K stimulation in CCM lesions

Several nongenetic factors may also contribute to the occurrence of CCM lesions. The number of CCM lesions expands in patients over 50 years of age (137). In these patients, increasingly hypoxic conditions during aging apparently fuel angiogenic signaling, thus contributing to the formation of vascular lesions. Similarly, in murine EC-specific knockouts of *Ccm* genes, lesions form most frequently in cerebellar and retinal vessels, two vascular beds undergoing active angiogenesis at postnatal stages (138), which is mediated by hypoxic conditions. Increased angiogenesis signaling is a hallmark feature of CCMs (129, 139–144). For instance, the loss of CCM3 increased the growth and microvascular density of a glioblastoma xenograft. This was triggered by the secretion of soluble factors, including VEGF, which mediates an activation of AKT (145). Enhanced angiogenic signaling may also trigger an overactivation of PI3K signaling together with its downstream targets AKT and mTOR. In fact, AKT and mTOR activities are strongly enhanced in *Ccm* LOF- and *Klf4* GOF-transgenic murine models (24). This study also revealed that an overstimulation of PI3K resulting from EC loss of *Pten* had similar effects as a genetic third hit. Therefore, PI3K not only triggers the formation of vascular anomalies when oncogenic GOF mutations render its signaling pathological, but also when there is an PI3K-independent overstimulation that turns PI3K signaling pathological (24).

The EC-specific murine knockout of *Ccm3* resulted in enhanced TIE2 signaling (146), which was triggered by an increased secretion of its low-affinity ligand ANG2 (147). ANG2 secretion antagonizes the blood vessel–stabilizing ANG1 and destabilizes EC junctional integrity (148, 149). This relationship underlies molecular similarities between the small brain capillary disease CCM and slow-flow VMs caused by GOF variants of *TEK* (150, 151).

## PI3Kα in VMs

VMs have been associated with GOF somatic mutations in *TEK* (74, 77, 78), accounting for up to 60% of sporadic VMs. These mutations cause an aberrant activation of PI3K/AKT signaling in a ligand-independent manner (116, 152, 153). Consequently, their activation directly affects blood vessel stability. Overactivation of the PI3K/AKT pathway downstream of *TEK* GOF variants stimulates mTOR signaling (reviewed in ref. 154), which causes changes in EC morphology (116) and overgrowth phenotypes in lymphatic and venous vessel beds (114, 115, 117, 155). Somatic mutations in *PIK3CA* were identified in another 25% of sporadic VMs (116, 152, 153), supporting the notion that TIE2 mainly signals through PI3Kα in this context. Strikingly, driving heterozygous *Pik3ca^H1047R^* within the murine developing vasculature using the *TIE2* promoter was sufficient to cause vascular remodeling defects, likely due to an overgrowth of endothelium (156). The co-occurrence of *TEK* and *PIK3CA* mutations in VMs is rare. Yet, in a few cases, both mutations have been identified in the same patient (157, 158). Nevertheless, it remains unknown whether both mutations occur in the same cell or whether lesions are genetically heterogenous. Possibly, *TEK* and *PIK3CA* mutations may arise subsequently within the same cell, implying that VMs may also develop upon second-hit events. In fact, germline mutations in *TEK* result in a weak autophosphorylation of the receptor, and, strikingly, a somatic second hit in the same gene is required to cause the typical multifocal and small-sized lesions in inherited cutaneomucosal VMs (150, 151) (reviewed in ref. 89). Kobialka and colleagues recently found that active angiogenesis is required for *Pik3a^H1047R^*-driven vascular malformations to occur (27). Thus, when mutations occur congenitally, focal lesions initially grow in size in tune with general body growth. Yet such focal lesions remain mostly quiescent with a low proliferating rate throughout adulthood. However, the presence of angiogenic stimuli can strongly stimulate proliferation in the affected vasculature and drive the growth rate of lesions. Interestingly, the levels of PI3K/AKT activation largely depend on TIE2 variants (153). Thus, it is tempting to speculate that PI3K-related vascular disease outcomes are determined by the severity of *TEK* mutations, which can trigger variable magnitudes of aberrant PI3K/AKT activation and abnormalities.

## Excessive PI3K signaling contributes to AVMs in HHT

In contrast to venous or capillary ECs, arterial ECs apparently detain a safeguarding mechanism against PI3K-triggered lesions. However, such a model was called into question with the surprising discovery that excessive PI3K/AKT signaling also contributes to the etiopathology of fast-flow AVMs (64), which are a hallmark of the inherited vascular disorder HHT. HHT pathogenesis involves monoallelic germline LOF mutations in the genes encoding the receptors activin-like kinase 1 (ALK1) (159) and the auxiliary coreceptor endoglin (ENG) (160) or the transcriptional effector SMAD4 (161), which are components of the canonical BMP9/10 signaling pathway (Figure 3).

Studies in murine HHT disease models demonstrated that an excessive activation of the PI3K/AKT pathway in nonarterial regions of the vasculature causes AVM formation (65, 162–165). The pharmacological inhibition using pan-inhibitors for PI3K or genetic inactivation of *Akt1* suppressed AVM formation in these murine HHT models. In addition, the inhibition of excessive mTOR or YAP/TAZ signaling, downstream of PI3K/AKT, suppressed or improved AVM formation (65, 162–164, 166). The pathological relevance of this finding was further substantiated by the detection of increased PI3K/AKT signaling in patients with HHT (66, 167). Currently, it is unknown which PI3K isoforms mediate such an excessive activation of AKT within AVMs. Yet, genetic studies with an heterozygous inactivation of *Pik3ca* prevented an *Alk1*-induced vascular hyperplasia within the retina (66), pointing at PI3Kα as an important player in AVM pathogenesis. Whether PI3Kα is the sole isoform involved in AVM pathogenesis remains an exciting question for future research. These findings also emphasize similarities in the molecular mechanisms that are shared in the etiology of AVMs, VMs, and CCMs.

Strikingly, a recent study with a murine EC-specific *Smad4*-knockout HHT model revealed that the FSS-induced KLF4/TIE2 overactivation in AVMs is upstream of PI3K/AKT signaling. These findings suggest a multifactorial regulation of PI3K/AKT by SMAD4 signaling. SMAD4 signaling restricts the activation of PI3K/AKT via a negative feedback loop involving PTEN (65, 164). In addition, SMAD4 acts upstream in defining the FSS set point, which limits the expression levels of KLF4/TIE2, which in turn affects the activation of the PI3K/AKT pathway.

These findings point to a surprising crosstalk between the HHT fast-flow and the slow-flow PI3K/AKT/mTOR pathway, hinting at the cellular origin of affected ECs in slow-flow venous and capillary beds from which these AVMs arise and in which the PI3K/AKT pathway plays a critical role during stages of AVM formation. Therefore, these fast-flow pathologies may originate within slow-flow regions of the vasculature at stages when PI3K signaling becomes excessively activated. These findings imply common cellular and molecular features of fast-flow with slow-flow vascular lesions and further support the concept that many vascular malformations arise due to excessive PI3K/AKT/mTOR activation, which leads to a permanent and chronic remodeling of nonarterial ECs.

Similar to some other vascular anomalies, including CCMs, AVM formation in HHT occurs in a characteristic spatial-temporal manner with a somatic mutation (second-hit mechanism) occurring at the second allele within the same germline-mutated gene leading to a biallelic LOF (168). The late appearance and focal distribution of AVM lesions to only selected vascular beds suggests that a loss of heterozygosity of HHT-associated genes is required but not sufficient to cause AVMs. Apparently, and similar to the disease progression in CCM and cancer, an additional third hit is required to initiate the disease progression. It has been proposed that locally high levels of VEGF, injury-mediated inflammation, or increased FSS are among the third hits contributing to AVM formation (169–172). Increasing evidence now points at an overactivation of the PI3K/AKT pathway in these instances (reviewed in refs. 71, 157, 173).

There is compelling evidence that the BMP9/10 signaling pathway and FSS act in a feedback loop to maintain EC quiescence and vascular homeostasis. Under physiological conditions, high laminar FSS potentiates the engagement of ALK1-ENG receptor complexes causing an activation of SMAD-1/5/8 signaling, which contributes to vascular stability (174, 175). Conversely, low laminar FSS potentiates BMP9-mediated SMAD-2/3 activation, which induces inward remodeling characterized by a reduction in vessel caliber (176). In turn, BMP9/SMAD4 sets the FSS set point by restricting activation of the flow-induced KLF4/TIE2/PI3K/AKT pathway, which limits EC remodeling events. This also maintains physiological FSS-mediated cell cycle arrest and arterial identity (64). Thus, exacerbated PI3K/AKT activity within AVMs is driven by both an inhibition of PTEN (65, 66, 164) and an increased activation of KLF4/TIE2 triggered by FSS (64). How BMP9 restricts FSS-mediated KLF4/TIE2/PI3K/AKT signaling to maintain ECs in a quiescent state remains unknown. The mechanism that drives an activation of KLF4 upon FSS stimulation does not involve the mechanotransduction receptor complex comprising PECAM, VE-cadherin, and VEGFRs (64). Therefore, excessive stimulation of KLF4 in AVMs may be mediated through ERK5 activation in a manner similar to KLF4 activation in CCMs (124, 125) (Figure 3), and this may increase PI3K/AKT signaling. Intriguingly, in the slow-flow vascular malformation CCM, KLF4 transcriptionally activates increased BMP6-mediated Smad2/3 pathway signaling, which causes an endothelial-mesenchymal transition (127, 177). Taken together, these studies demonstrate the surprising interconnection of various biomechanical signaling pathways that converge onto an excessive overstimulation of the PI3K/AKT pathway. Consequently, this pathway activation can contribute as a third hit in the pathogenesis of fast-flow AVMs, yet another type of vascular malformation.

## Fast-flow malformations in PHTS

Fast-flow AVMs can present with unique lesions (as in HHT) and, when occurring sporadically, can be part of a combined vascular syndrome such as the capillary malformation–AVM (CM-AVM) syndrome. Alternatively, they may present as the vascular feature of a complex syndrome of the *PIK3CA*-related tissue overgrowth spectrum (PROS), as in PHTS or Proteus syndrome.

The CM-AVM syndrome is caused by germline or inherited LOF variants in two genes, RAS P21 protein activator 1 (*RASA1*) (178, 179) and ephrin receptor B4 (*EPHB4*) (180), accompanied by an additional somatic variant acting as a second hit (181). Interestingly, somatic mutations in genes in the RAS/MAPK pathway leading to overactivation of this pathway were identified also in sporadic AVMs, with *MAP2K1* mutations in extracranial lesions and somatic activating mutations in *KRAS* being prevalent in brain AVMs (bAVMs) (182–184). Activated KRAS in bAVM lesions resulted in an overactivation of the major downstream MAPK/ERK and PI3K/AKT signaling pathways. Noteworthy, only specific inhibition of MAPK ameliorated the bAVM outcome in the context of *Kras* mutations, suggesting that inhibition of the MAPK/ERK pathway might be a promising target for a subset of bAVMs. This finding alerts us that the term RASopathies does not encompass all fast-flow malformations and should be applied only to fast-flow lesions with a RAS pathway activation. Therefore, despite the fact that HHT-related AVMs present as fast-flow lesions, based on their molecular outcome, they should be classified as PIKopathies (see above) (Figure 3).

Approximately 30% of patients with PHTS present vascular lesions, predominantly AVMs that are localized to the extremities (185). Similar to other malformations, PHTS-related AVMs are not apparent until patients reach an older age, emphasizing the requirement for additional genetic and/or environmental hits for disease progression. Yet, it remains to be determined which pathogenic *PTEN* variants trigger lesion formation. During embryonic development, Pten has an antiangiogenic role in zebrafish (186). In mice, EC loss of *Pten* results in cardiac failure and severe hemorrhaging during early embryogenesis (187), while postnatally, PTEN is required for NOTCH-mediated cell cycle arrest in retinal stalk cells (188). In this context, both catalytic and noncatalytic activities of PTEN contribute to this function, providing evidence for dual physiological functions. PTEN is also required for BMP9/10-mediated vessel patterning (164). Therefore, PTEN is regulated by NOTCH, BMP, and FSS. Why *Pten* LOF mutations predominantly cause AVMs rather than VMs remains a puzzling question for future interrogations.

## Future directions

Recent findings demonstrate that not only slow-flow, but also some fast-flow, vascular anomalies involve an overstimulation of PI3K/AKT signaling. A number of studies have identified PI3K as a central node that integrates physiological responses of ECs to the trigger of biomechanical forces due to FSS. The present research implies that remodeling of mature vessel beds results from a pathophysiological resetting of a PI3K/AKT-dependent FSS set point. This induces a loss of EC dormancy and quiescence and causes vascular instability and disease. In agreement with such a model, levels of PI3K activation correlate with the aggressiveness and severity of lesion formation. In some vascular anomalies, PI3Kα GOF variants are the main driver of disease severity, i.e., when occurring as a third genetic hit to activate dormant CCM lesions (24). In VMs, the severity of different GOF variants of the angiogenesis receptor TIE2 leads to different outcomes (153), and this may depend on the levels of PI3K activation. Therefore, understanding this pathway in a wide range of vascular malformations comes with the opportunity of normalizing pathological signaling in affected ECs because the PI3K/AKT signaling pathway is highly druggable.

Yet, we currently lack a good understanding of how different types of FSS and patterns of blood flow impact the overactivation of PI3K signaling in vascular anomalies. Molecular studies and a systematic characterization of PI3K/AKT/mTOR activities in animal disease models and tissue culture systems with well-defined FSS conditions are urgently needed to address these fundamental questions in the future. Although vascular cells harbor the same basic machinery, different vessel beds respond to blood flow in different ways. It will be an exciting avenue for future translational endeavors to test whether targets of dysregulated PI3K/AKT signaling in diseased cells can be normalized pharmacologically by normalizing excessive signaling in response to flow. Such systematic approaches will lay the groundwork for understanding whether specific drugs can be optimized to treat particular slow- versus fast-flow regions of the vasculature and whether combinatorial drug treatments may be an option.

Currently, the standard therapy for vascular anomalies is surgical resection or sclerotherapy. However, such approaches are not feasible when the affected vasculature is too extensive in size or when malformations are located in inoperable regions of the body such as the spinal cord. Instead, pharmacological therapies of patients with GOF mutations in the TIE2/PI3K/AKT/mTOR pathway hold a great promise and have been fueled by studies using repurposed anticancer drugs. The central role of PI3K/AKT/mTOR signaling in many types of cancer has spurred research in that direction with different classes of such inhibitors currently being tested in clinical trials. These involve pan- and isoform-specific PI3K as well as dual PI3K/mTOR inhibitors (reviewed in ref. 189). The results of these studies regarding the efficacy, safety, and specificity of drugs will be of great interest also for the treatment of vascular anomalies. Several patients with PROS were treated successfully with the PI3K inhibitor alpelisib, which reduced their symptoms (25). Targeting AKT with miransertib (ARQ 092), an allosteric and selective pan-AKT inhibitor, has been used effectively in the treatment of patients with Proteus syndrome (190) and PROS (191, 192). Rapamycin is an inhibitor of the mTOR1 complex, and its efficacy in suppressing vascular anomalies has been demonstrated in a number of clinical studies (193, 194). However, rapamycin showed less efficacy in the treatment of AVMs compared with its effects on slow-flow vascular anomalies (195–197). Future pharmacological interventions will be optimized based on what has been learned about genetic causes and molecular mechanisms of these diseases in relation to the flow conditions of affected vessel beds. Such approaches will certainly help to lessen the manifestation of a plethora of vascular anomalies, including VMs, CCMs, HHT-AVMs, and others. Future endeavors will address this promising possibility.

## Figures and Tables

**Table 1:**
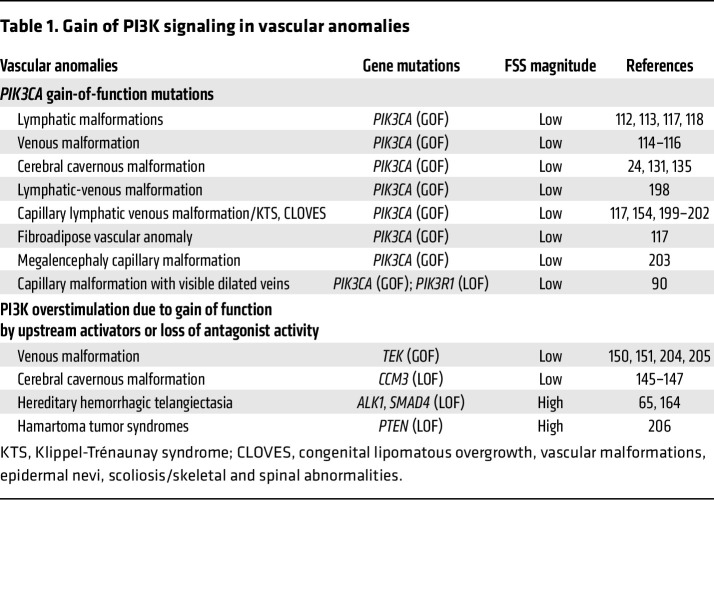
Gain of PI3K signaling in vascular anomalies

## Version 1 05/15/2024

Electronic publication
